# Opening the file drawer: Unexpected insights from a chytrid infection experiment

**DOI:** 10.1371/journal.pone.0196851

**Published:** 2018-05-09

**Authors:** Allison Q. Byrne, Thomas J. Poorten, Jamie Voyles, Craig K. R. Willis, Erica Bree Rosenblum

**Affiliations:** 1 Department of Environmental Science, Policy & Management, University of California, Berkeley, California, United States of America; 2 Department of Biological Sciences, University of Idaho, Moscow, Idaho, United States of America; 3 Department of Biology and Centre for Forest Interdisciplinary Research, University of Winnipeg, Winnipeg, Manitoba, Canada; University of South Dakota, UNITED STATES

## Abstract

Infection experiments are critical for understanding wildlife disease dynamics. Although infection experiments are typically designed to reduce complexity, disease outcomes still result from complex interactions between host, pathogen, and environmental factors. Cryptic variation across factors can lead to decreased repeatability of infection experiments within and between research groups and hinder research progress. Furthermore, studies with unexpected results are often relegated to the “file drawer” and potential insights gained from these experimental outcomes are lost. Here, we report unexpected results from an infection experiment studying the response of two differentially-susceptible but related frogs (American Bullfrog *Rana catesbeiana* and the Mountain yellow-legged frog *Rana muscosa*) to the amphibian-killing chytrid fungus (*Batrachochytrium dendrobatidis*, Bd). Despite well-documented differences in susceptibility between species, we found no evidence for antibody-mediated immune response and no Bd-related mortality in either species. Additionally, during the study, the sham-inoculated *R*. *catesbeiana* control group became unexpectedly Bd-positive. We used a custom genotyping assay to demonstrate that the aberrantly-infected *R*. *catesbeiana* carried a Bd genotype distinct from the inoculation genotype. Thus *R*. *catesbeiana* individuals were acquired with low-intensity infections that could not be detected with qPCR. In the Bd-inoculated *R*. *catesbeiana* treatment group, the inoculated genotype appeared to out-compete the cryptic infection. Thus, our results provide insight into Bd coinfection dynamics, a phenomenon that is increasingly relevant as different pathogen strains are moved around the globe. Our experiment highlights how unexpected experimental outcomes can serve as both cautionary tales and opportunities to explore unanswered research questions. We use our results as a case study to highlight common sources of anomalous results for infection experiments. We argue that understanding these factors will aid researchers in the design, execution, and interpretation of experiments to understand wildlife disease processes.

## Introduction

Emerging infectious diseases are increasingly recognized as a challenge for wildlife conservation [1–3]. To understand host-pathogen interactions in natural systems, researchers often turn to laboratory infection experiments (e.g., [4–9]). Although infection experiments have tremendous value for understanding disease processes, there are potential pitfalls to experimental approaches. Disease outcomes are influenced by interactions among host, pathogen and environment [2]. The complexity of these interactions can affect the repeatability of infection experiments among laboratories or even within the same laboratory over time. This, in turn, can lead to ambiguous experimental results that are difficult to interpret, apply to disease management and conservation, and publish.

The "file drawer" problem–where non-significant or ambiguous results are less likely to be published in the scientific literature [10]–is a longstanding issue in biological research. Not only are journals often biased against non-significant results [11,12], but researchers may be less likely to submit results that are inconsistent with past work and/or difficult to interpret [13]. The file drawer problem in wildlife disease research is a potentially significant obstacle to progress. Differences among studies and contradictory results have the potential to reveal biologically important insights about disease systems via carefully informed interpretation and meta-analyses (e.g., [14]). Contradictory results can also reveal systemic problems in the design of experiments and be used to help improve experimental design.

Laboratory infection experiments have been essential in advancing our understanding of one of the most devastating wildlife diseases, amphibian chytridiomycosis. Chytridiomycosis is an emerging infectious disease caused by the fungal pathogen *Batrachochytrium dendrobatidis* (Bd) [15]. Infection experiments have revealed complex interactions among Bd, host defenses, and environmental conditions (reviewed in [16]). For example, experimental infections have been used to quantify Bd transmission rates [17], describe how temperature affects disease outcomes [18], document host transcriptional response to infection [19], and investigate differences in susceptibility among host species and differences in virulence among Bd isolates [20,21].

Here, we discuss results from a Bd infection experiment designed to investigate species-level differences in two related, yet differentially susceptible amphibian species. The American bullfrog (*Rana catesbeiana*) and the Mountain yellow-legged frog (*Rana muscosa*) shared a most recent common ancestor ~50.6 million years ago [22,23]. *R*. *catesbeiana* is tolerant to Bd and exposed individuals usually maintain low infection loads [24]. Additionally, *R*. *catesbeiana* is an invasive species in many parts of the world and has been implicated in spreading Bd to novel areas [25,26]. In contrast, *R*. *muscosa* has suffered population declines across its range due to Bd [27,28], and many studies have documented high susceptibility to Bd in this species (e.g., [5,18]). However, recent studies indicate that some *R*. *muscosa* populations are rebounding while maintaining enzootic Bd infections [29].

Given our objective to investigate responses to Bd infection in two differentially susceptible frog species, we measured several response variables following experimental inoculation with Bd. First, we tracked Bd load using a qPCR assay from skin swabs. Second, we measured percent change in body mass as a proxy for disease-related changes in body condition. Third, we used an ELISA (enzyme-linked immunosorbent assay) to measure antibody-mediated immune responses to Bd infection, as this could be an underlying mechanism for resistance or tolerance. We hypothesized that *R*. *catesbeiana* would be more resistant to Bd infection, show lower Bd loads, less negative change in body mass, less mortality, and more antibody-mediated immunity than *R*.*muscosa*. However, we were surprised to find no mortality in the highly-susceptible *R*. *muscosa* and no evidence of antibody-mediated immunity in either species. Moreover, during the experiment one of our control groups became unexpectedly Bd-positive. Our study was initially relegated to the file drawer because of these anomalous results, but after developing a new Bd genotyping assay, we were able to diagnose the source of one important anomaly and gain new insights into disease dynamics in this system.

While our experiment exemplifies some of the pitfalls that can arise during infection experiments, it also highlights the importance of publishing atypical results from infection experiments to advance both methodological and ecological investigations of disease systems. Thus, in addition to reporting what were initially anomalous results of one experiment, we review the most important factors that could influence infection experiments and lead to ambiguous or confusing results in this and other wildlife disease systems.

## Methods

We transported adult *R*. *muscosa* in separate containers from San Francisco State University to University of Idaho in April 2011 (n = 37, mean mass: 9.60g ± 0.63g). We ordered small adult *R*. *catesbeiana* (n = 40, mean mass: 109.79g ± 2.20g) from Rana Ranch Bullfrogs (Twin Falls, Idaho) and shipped them to the University of Idaho in one large container in March 2011. Upon arrival, we moved all frogs into individual plastic containers in temperature (20°C), humidity (50%) and light (12L/12D) controlled facilities. We fed frogs vitamin-dusted crickets (5-week old, Fluker Farms, Port Allen, LA) *ad libitum* twice per week. We changed their water (approximately 250 ml, tap water) twice a week until experimental exposures began, then we replaced tap water with 20% Holtfretter’s solution (in mM: NaCl (6), KCL (0.06), CaCl2 (0.09), NaCO3 (0.24), pH 6.5, 250 ml). We placed the containers in a level position so that water covered the bottom. All methods used for this study were approved by the IACUC at the University of Idaho (University of Idaho AUP 2008–55).

For the Bd inoculation we selected isolate CJB5-2 because it was highly pathogenic in previous laboratory exposure experiments (C. Briggs, M. Toothman *pers comm*). This isolate was originally collected from a diseased *R*. *muscosa* frog caught at Barrell Lakes, CA. We cultured the isolate on tryptone/gelatin hydrolysate/lactose (TGhL) agar plates with antibiotics [15] and then maintained the culture in TGhL broth at 4°C, passaging every 2 to 3 months. Ten days prior to exposure, we incubated Bd for 4 days on agar plates maintained at 23°C. We harvested Bd zoospores for animal infections using dilute salt solution (in mMol: KH_2_PO_4_ (1.0), CaCl_2_ (0.2), MgCl_2_ (0.1)) and quantified zoospores using a haemocytometer [30]. We randomly separated frogs into four different treatment groups: Bd-inoculated *R*. *catesbeiana* (n = 20), sham-inoculated *R*. *catesbeiana* (n = 20), Bd-inoculated *R*. *muscosa* (n = 18), and sham-inoculated *R*. *muscosa* (n = 19). We inoculated frogs with 1x10^6^ zoospores in 30ml of 20% Holtfretter’s solution (following [31]). For the control frogs, we sham-inoculated frogs with a bath comprised of 20% Holtfretter’s solution and dilute salt solution collected from agar plates without Bd cultures. After 24 h in their respective exposure solutions, we moved frogs to freshly-cleaned plastic containers with 250 ml 20% Holtfretter’s solution. Following exposures, we monitored the frogs daily for clinical signs of chytridiomycosis including lethargy, inappetence, cutaneous erythema, irregular skin sloughing, and abnormal posture. We fed frogs and changed the Holtfretter’s solutions twice weekly for the duration of the experiment. We weighed frogs initially and every week throughout the experiment. We used Student’s t-tests to compare mean percent change in weight at different sampling points over time (repeated measures type) and between treatment groups (independent measures type).

We collected skin swab samples before inoculation and every week for the 10-week duration of the experiment. Collecting skin swab samples is a non-invasive technique that involves rubbing a sterile cotton swab (Medical Wire & Equipment, Corsham, UK) over the ventral surfaces and digits [32]. We analyzed skin swab samples for Bd presence using quantitative polymerase chain reaction (qPCR) for Bd using standard protocols (Taqman real-time qPCR assay, 30), with standards provided by Alex Hyatt (Australian Animal Health Laboratory, Geelong, Australia). We analyzed our Bd DNA extractions in duplicate and considered a well positive if zoospore genetic equivalent > 0.01.

We collected blood samples before exposure and over the course of infection to evaluate for the presence of Bd-specific antibodies using an ELISA (enzyme-linked immunosorbent assay) as done in [33] (see S1 File). Briefly, we collected blood samples (< 1% frog weight) using a heparinized syringe and needle via cardiac puncture after anesthesia by shallow immersion in 0.1% MS222 solution (tricaine methanesulfonate, Sigma Chemical) buffered with sodium bicarbonate (0.4%, Sigma Chemical), which does not kill Bd [34]. Although anesthesia for blood sampling can be stressful for amphibians, it does not alter blood electrolyte concentrations [35]. Nonetheless, we limited blood sample collections to 30 and 65 days post-inoculation to minimize the stress to experimental animals. We isolated blood serum by centrifugation and stored at -80°C until use in the ELISA. We used Student’s t-tests to compare mean antibody concentrations (measured as optical density at 450 nm using a Bio-Rad 680 Microplate Reader) at different sampling points.

After the experiment was concluded we genotyped the Bd present on 16 of the skin swab samples (2 Bd-exposed *R*. *catesbeiana*, 8 control *R*. *catesbeiana*, 6 Bd-exposed *R*. *muscosa*) collected during weeks 2, 4, and 8 of the experiment using a custom genotyping assay (see [36]). Briefly, this assay uses microfluidic multiplex PCR to amplify select regions of the Bd genome using DNA collected from swab samples. We concatenated sequences from 90 nuclear loci for all 16 swab samples and 26 Bd isolates. The Bd isolates have previously characterized phylogenetic relationships (see [37]) and were used to contextualize the phylogenetic relationships of the skin swab samples. We aligned the concatenated sequences using MUSCLE v3.8 [38] and visually checked the alignments for errors. We then selected the best-scoring maximum likelihood tree from 1000 bootstrap replicates using the RAxML plugin v.2.0 [39] in Geneious v.10.0.7 [40]. All sequences and data files are deposited in the DRYAD database (https://doi.org/10.5061/dryad.f127gp0).

## Results

**Fig 1:**
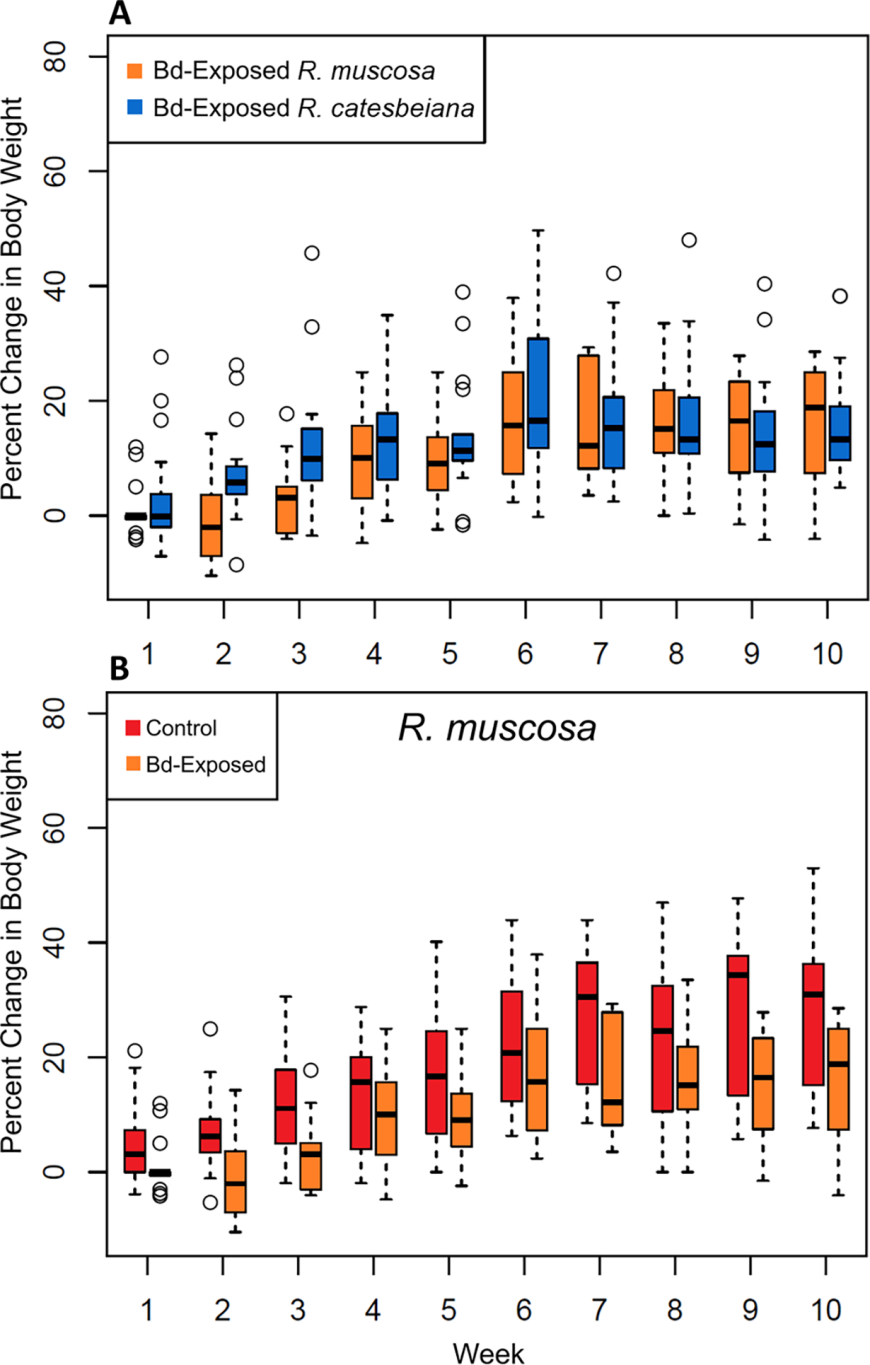
Percent change in body weight for each species throughout the experiment. (A) Box plot showing percent change in weight (from day 0) over the 10-week course of the experiment for *R*. *catesbeiana* Bd-exposed and *R*. *muscosa* Bd-exposed treatment groups. (B) Percent body weight change for *R*. *muscosa* Bd-exposed and control treatment groups.

We found no differences between the two focal species–or the treatment groups–in mortality, percent change in body weight, or circulating antibody levels. In fact, we observed no Bd-related mortality in any of our treatment groups. All *R*. *muscosa* Bd-exposed individuals survived for the duration of the experiment, although three *R*. *muscosa* in the control group died during week 6 for unknown reasons. All *R*. *catesbeiana* individuals in the control group survived the duration of the experiment, while one individual in the *R*. *catesbeiana* Bd-exposed group was euthanized during week 6 because of a broken leg. There were no significant differences in overall percent body weight change between the *R*. *muscosa* control and Bd-exposed individuals (two-tailed t-test p-value 0.12, Fig 1B) or between the Bd-exposed *R*. *muscosa* and the Bd-exposed *R*. *catesbeiana* groups (two-tailed t-test p-value 0.39, Fig 1A). For the ELISA, all blood serum samples collected from both species across all three time periods (Pre-infection, 30 days post-infection, 65 days post-infection) were not significantly different from the negative control (α = .01) and were significantly different from the positive control (one-tailed t-test p-value < .01, Table A in S1 File). Thus, neither species produced detectable Bd-specific antibodies at any point during the experiment.

**Fig 2:**
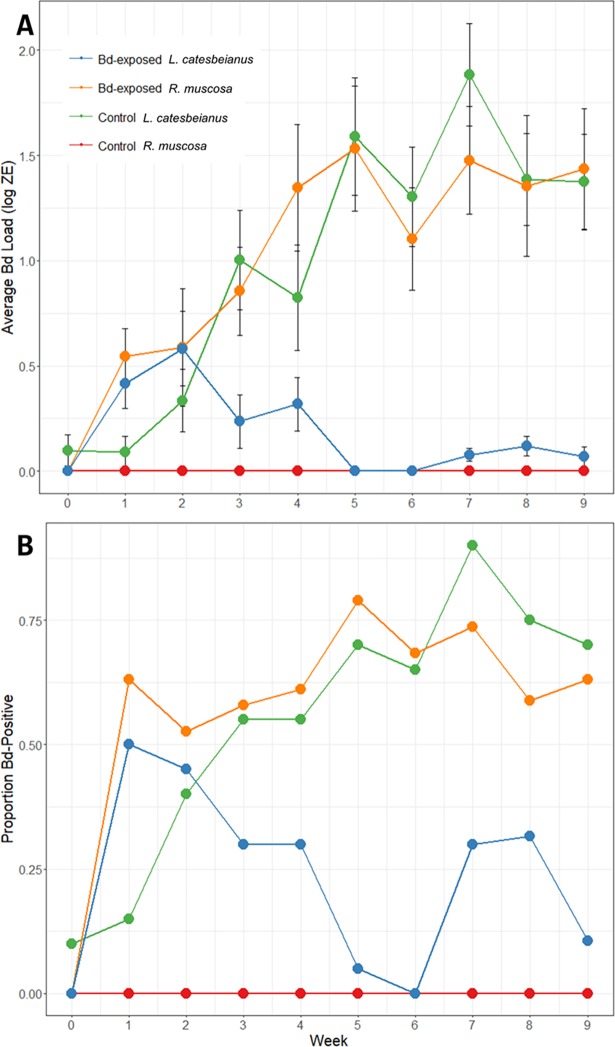
Bd prevalence and intensity across all treatment groups throughout the experiment. (A) Average Bd load, measured as log Bd zoospore equivalents (ZE) from qPCR of skin swabs, for each treatment group over the duration of the experiment. Error bars are standard error of the mean. (B) Proportion of individuals in each treatment group that were positive for Bd (ZE > 0.01) every week for the duration of the experiment.

**Fig 3:**
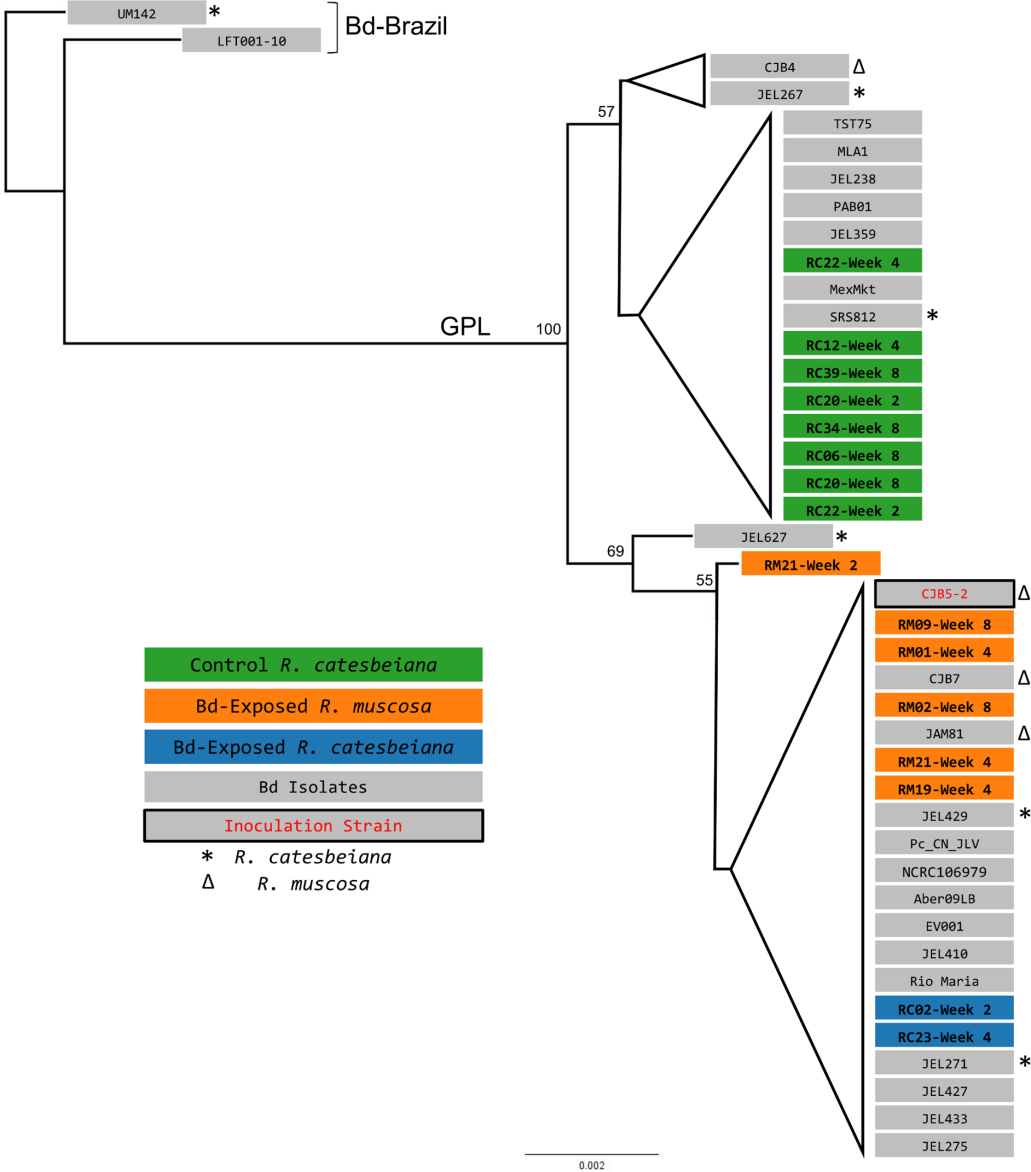
Phylogenetic comparison of Bd DNA collected via swabs during the experiment. Best-scoring maximum likelihood tree for 26 Bd isolates and 16 skin swab samples from 90 concatenated nuclear loci (12,080–12,103 bp). Bootstrap values above 50% (out of 1000 replicates) are shown and branches reproduced in less than 50% of bootstrap replicates are collapsed. Tree was rooted at Bd isolate UM142 based on previous phylogenetic characterization of the isolates used in this study [37]. Major Bd clades (Bd-Brazil and GPL) are indicated on the phylogeny. The Bd isolate used for inoculation in this study is indicated by a black box with red text. Bd cultures originally isolated from *R*. *catesbeiana* individuals are denoted with a * and Bd cultures originally isolated from *R*. *muscosa* are denoted with a Δ.

We did find differences between species and treatment groups for infection prevalence and intensity (Fig 2). As expected, control (sham-inoculated) *R*. *muscosa* were negative for Bd for the duration of the experiment. Exposed *R*. *muscosa* exhibited increasing Bd loads over time, but had lower Bd loads than reported in most previous *R*. *muscosa* infection experiments (e.g., [5,41]). In contrast, exposed *R*. *catesbeiana* typically cleared (or nearly cleared) their Bd infections after the first month. However, we unexpectedly discovered that some of the individuals in the *R*. *catesbeiana* control group (sham-inoculated) were positive for Bd. Although initial (pre-inoculation) skin swabs from all frogs were negative for Bd, the first post-inoculation qPCR results (day 0 of the experiment) were unexpectedly positive for this control group. Over the course of the experiment Bd prevalence and intensity (measured as Bd Zoospore equivalents, ZE) in this control group increased and were comparable to the Bd prevalence and intensity seen in the *R*. *muscosa* Bd-exposed group (Fig 2). To further understand how individuals in the *R*. *catesbeiana* control group became Bd-positive, we genotyped 16 of the Bd-positive skin swab samples (Fig 3). We found that the *R*. *catesbeiana* control frogs were not infected with the Bd isolate that we used for inoculations (CJB5-2), but rather a significantly diverged Bd isolate. The Bd on swabs collected from *R*. *catesbeiana* and *R*. *muscosa* Bd-exposed frogs was positioned as expected in the phylogeny–forming a well-supported cluster with the inoculation isolate (CJB5-2, Fig 3). However, the mystery isolate was found in a distinct subclade within the Bd-GPL clade (Global Panzootic Lineage [42], Fig 3).

## Discussion

Although researchers aim to control as many covariates as possible among experiments, there are many decisions to be made when planning infection experiments and many possible explanations for inconsistent and contradictory results. In our study, we found two unexpected results. First, all of the Bd-exposed *R*. *muscosa*, a species known to be susceptible to Bd [27], survived for the duration of the experiment with relatively-low Bd loads. Second, the control (sham-inoculated) *R*. *catesbeiana* became Bd-positive and individuals in this group showed increasing Bd prevalence and intensity over the course of the experiment.

Initially we could not explain either of these results until we reexamined two key factors in our experiment. First, using a new genotyping assay we developed [36], we determined that the *R*. *catesbeiana* control frogs were infected with a distinct Bd genotype that had not previously been present in our lab. Therefore, the *R*. *catesbeiana* likely arrived with low-intensity infections that could not be detected with qPCR from swab samples. Second, after interrogating lab notebooks and culture records–and given what we know about Bd attenuation from lab culturing practices [43]–we hypothesize that the Bd isolate we used may have attenuated in virulence over time, facilitating the survival of the susceptible *R*. *muscosa*.

Investigating our own anomalous experimental results motivated us to reflect on general explanations for unexpected results in infection experiments. Here we discuss several common (and non-mutually exclusive) pitfalls researchers may face when conducting infection experiments. We use the results of our experiment as a case study, but our aim is to help researchers avoid common pitfalls and stimulate publication–and meta-analyses–of other ambiguous results that may shed light on the biology of host-pathogen interactions.

### Experimental design effects

Perhaps the most obvious potential source of variation among experiments is inconsistency in how experiments are designed and in the mechanics of how they are conducted. Often infection experiments–even those on the same disease system–cannot be replicated identically. Equipment, protocols, personnel, and availability of animal and pathogen stocks will rarely be identical across laboratories, or even within a single laboratory over time. For example, in studies of amphibian chytridiomycosis, even basic decisions such as the source of water used for inoculations [44], or the type of gloves used [45] can affect the outcome of experiments. Decisions and factors like these may seem minor during the planning stage and are rarely reported in enough detail to understand if they may explain inconsistent results.

As in all experimental science, sampling error and sample size can be major driver of inconsistency among experiments. Experiments may be limited in sample size due to financial, ethical, or infrastructure constraints, particularly if species of interest are endangered or imperiled due to the disease (e.g., [6,46,47]). Stochastic effects are more likely to cause spurious results when sample sizes or experimental replicates are low. Moreover, when sampling from wild populations, the individuals captured may not represent the population at large, particularly when it is difficult to sample randomly (for example, sampling egg masses can bias toward highly related individuals; sampling based on conspicuousness can bias toward behaviorally bolder individuals). Therefore, even if host individuals–or pathogen isolates–are drawn from the same population for a series of experiments, sampling error could lead to differences in outcomes.

The sampling design used in our experiment illustrates some of these pitfalls. Due to ethical and practical considerations working with an endangered species, our sample size was limited to ~20 individuals per treatment group. Moreover, we sourced frogs from a single population for each species and selected a single Bd isolate to use for inoculations. We also conducted experiments under a single common garden environmental regime. Given practical limitations in sample size, these choices were reasonably well matched to our primary biological question (i.e., are there differences in susceptibility across species?). However, a more complete understanding of patterns of intra- and inter-specific variation in susceptibility would require tests with multiple source populations and multiple Bd isolates under multiple environmental conditions.

Finally, it is critical to match the experimental design to the research questions of interest. Infection experiments are not always the best approach for addressing complex questions in disease systems because they are necessarily limited in their ability simulate natural environmental conditions, ecological, and evolutionary interactions (as discussed below). For example, lab experiments often assess host susceptibility based on infection with a single pathogen isolate under one or few environmental conditions and thus cannot truly predict how a species is likely to respond to a pathogen outbreak in the wild. Manipulating the right variables for the question and conducting explicit power analyses is critical during the design stage of infection experiments.

### Captivity effects

Emerging infectious diseases of wildlife often occur in species that are relatively poorly studied and difficult to maintain in captivity (e.g., insectivorous bats, large mammals, amphibians). In many cases, when disease studies are first undertaken, best practices for captive maintenance of host species in the laboratory have not yet been established. This creates the potential for large, un-reported variation in protocols among laboratories, as various groups develop their own husbandry practices.

In addition, despite best intentions to simulate key aspects of the biotic and abiotic context for infection, captivity can affect host, pathogen, and symbiont biology in dramatic ways. From the host perspective, changes in physiological stress, diet, activity levels, and social context all have the potential to alter host responses to pathogen exposure [48]. Moreover, some species (and individual animals) will acclimate to laboratory conditions more quickly than others. Thus, if wild-captured hosts are used for infection studies, time in captivity prior to infection could impact experimental results. Moreover, changes in host behavior in captivity can have significant impacts on disease outcomes. For example, exposure to heat can reduce Bd infection intensity in some amphibian species [49], and many species in the wild behaviorally thermoregulate in response to Bd infection (e.g., [50]). Thus, uniform temperatures in the lab can restrict behavioral thermoregulation and provide a poor understanding of a species’ ability to combat disease in nature.

From the pathogen perspective, laboratory conditions–like temperature and nutrient conditions–can affect pathogen survival, virulence and rates of reproduction [51–53]. From the symbiont perspective, biotic co-factors can be lost or altered in captivity. In the wild, symbionts can protect hosts from–or predispose hosts to–infectious disease [54,55]. Depending on laboratory maintenance practices, these biotic interactions can be perturbed. For example, hosts can lose beneficial microbial commensals when brought into captivity [56,57]. This is particularly important in amphibian systems where a wealth of studies document the role of skin-microbes in mediating the effects of Bd (e.g., [41,53,57]). Similarly, the standard practice of isolating pure pathogen cultures (often with broad spectrum antibiotics) can select against a range of cofactors important in the wild. The effects of captivity on host-pathogen interactions typically go undetected or unreported.

While both frog species in our study were kept under identical conditions during the experiment, they experienced different environments prior to arriving in our lab. The *R*. *catesbeiana* were obtained from Rana Ranch Bullfrogs (Twin Falls, Idaho), a commercial frog seller who advertise their product as “purpose bred bullfrogs that are raised from eggs in clean geothermal water under intensive aquaculture conditions” [58]. In contrast, the *R*. *muscosa* used in this study were raised from wild collected egg masses and shipped from captive populations at San Francisco State University. Differences in how these animals were raised, including previous exposure to stress (for example, exposure to temperatures outside of physiological optima, high population densities, and prior exposure to Bd) likely affected our experimental results. Additionally, both species used in this study are known to behaviorally thermoregulate [59,60]. Conducting our experiment in a single, homogenous thermal environment may not reflect natural Bd dynamics in these species. Most notably, our results indicated that the *R*. *catesbeiana* were exposed to a distinct Bd isolate before arrival in our lab. Additionally, all *R*. *catesbeiana* were shipped in a single container from their commercial source which may have facilitated the spread of Bd before they reached our lab.

The presence of a cryptic Bd infection in our *R*. *catesbeiana* individuals was surely a significant–and unexpected–biotic cofactor. Not only did we document an unexpected infection in our control *R*. *catesbeiana*, but we also found indirect evidence for competition between Bd isolates. Frogs were randomly assigned to treatment groups and individually housed throughout the experiment. Thus, we inoculated experimental *R*. *catesbeiana* with isolate CJB5-2 on top of an existing cryptic Bd infection. Our genotyping results suggest that CJB5-2 outcompeted the prior isolate in these lab-infected frogs, although sequencing Bd from additional late-stage infected *R*. *catesbeiana* would help confirm this hypothesis. Surprisingly, after two weeks the experimentally Bd-exposed *R*. *catesbeiana* exhibited *lower* Bd prevalence and intensity than both conspecific controls and experimentally exposed *R*. *muscosa*. What explains these patterns remains speculative. Bd is known to inhibit lymphocyte-mediated immune responses [61], and Bd isolates vary in their inhibitory capacity [62]. Moreover, high doses of Bd can trigger innate immune responses, as has been observed in *R*. *catesbeiana* larvae [63] and other amphibians [64]. Thus, it is possible that in our experiment there were differences in capacity for immune suppression between the Bd isolates or that the addition of a high-dose inoculum triggered a host immune response. Regardless, it remains an important observation that the only infected frogs that cleared their Bd infection in our experiment were those infected with two divergent Bd isolates.

Our experiment highlights the need for testing and treatment of experimental animals throughout their time in captivity and immediately prior to inclusion in an experiment. As much as possible, animals should be raised under controlled conditions and free from extraneous stressors. Rigorous tests for previous pathogen presence, such as serological tests, should be conducted to make sure animals collected in the wild are free from infection. Prolonged acclimation periods and multiple tests for pathogen presence can help reveal cryptic infections. Pre-treating all animals prior to conducting an infection experiment is one way to make sure all animals are uninfected, however some treatments themselves may cause stress (i.e. antifungal treatments, heat treatments) and have unintended effects on experimental results. Moreover, characterizing and comparing the microbial symbiont community among individuals, between treatment groups, and between captive and wild populations can help reveal the effects of captivity on biotic interactions and the effects of biotic interactions on infection outcome.

### Evolutionary effects

Laboratory conditions can affect not only the short-term behavior and physiology of individuals, but also the evolutionary trajectory of focal species. This is especially true for microbial pathogens that typically have short generation times, large population sizes, and fast mutation rates [65,66]. Microevolutionary changes in the pathogen can lead to rapid shifts in virulence, and there are numerous examples of virulence attenuation in pathogens maintained for multiple generations in the laboratory (reviewed in [67]). While there is less empirical evidence for evolution of hosts under experimental conditions, some hosts are known to exhibit rapid evolution of resistance or tolerance to pathogens (bacteria [68], insects [69]). If “survivors” from inoculation experiments are returned to a captive breeding colony–a practice which could be important when dealing with endangered species–artificial selection for resistance or tolerance could occur. Although evolving resistance in captive colonies may be considered a favorable outcome, it is still important to account for these evolutionary effects when interpreting experimental results and predicting disease outcomes in nature. Moreover, tolerance of captivity itself could lead to microevolution in a research colony that could influence traits affecting host susceptibility.

In addition to microevolution of host and pathogen populations independent of each other, there is pervasive evidence for coevolution of hosts and pathogens in natural disease systems [70]. Most relevant to infection experiments, hosts and pathogens from the same geographic area are likely to have close co-evolutionary relationships. Hosts can exhibit strain-specific resistance, and pathogens can show microgeographic adaptation to particular hosts [71]. Thus, infection outcomes might depend on whether a given host population is naïve to a particular pathogen strain–or conversely whether they share a co-evolutionary history [72]. Co-evolutionary dynamics could lead to inconsistent results across experiments, for example if the host and pathogen used in one experiment are geographically matched, while those used in a subsequent experiment are mismatched.

In our study, microevolution may have played a role in our unexpected results. The inoculation Bd isolate CJB5-2 was originally collected from an *R*. *muscosa* individual at Barrett Lakes, California, USA and was reportedly highly pathogenic in previous laboratory exposure experiments (C. Briggs, M. Toothman *pers comm*). However, we kept this isolate in culture in the laboratory at 4°C for months prior to being cryopreserved and subsequently revived to be used in our experiment. After revival, we kept the isolate at 4°C and grew it for a total of 42 days before passaging it. We then harvested zoospores from the passaged plates 10 days later. These details are important because maintaining Bd in laboratory conditions can cause rapid changes in virulence, depending on how and when isolates are passaged. For example, one study showed that passaging Bd once a month for a year caused Bd to attenuate in virulence and increased survival of a susceptible frog species [43]. The passage history of the isolate used in this experiment closely resembles the passage history of the attenuated isolate from [43] and points to pathogen attenuation as a possible explanation for *R*. *muscosa* survival, however additional experiments would be necessary to test this hypothesis.

In our study, the inoculation isolate was isolated from one of the study species, *R*. *muscosa*. Our rationale for using one Bd isolate was simply to minimize the number of experimental animals required–*R*. *muscosa* is endangered and doing a reciprocal experiment with a bullfrog isolate would have required twice as many individuals. Additionally, we chose an isolate that was thought to be virulent to increase our chances of detecting immune responses in both species. However, a reciprocal experimental design with Bd isolates from both species could provide additional insights into whether host-pathogen co-evolution has occurred.

Thus, it is important to consider the potential for co-evolutionary dynamics–and microevolution in the lab–to influence the outcome of infection experiments. Some laboratory practices can decrease the potential for accidental experimental evolution. For example. cryoarchiving pathogen isolates immediately after collection can reduce unintended laboratory microevolution [73,74], although cryoarchiving itself could have unintended consequences that still require evaluation. Regardless, the passage histories of pathogen isolates should always be reported, and results should be interpreted cautiously if these histories vary. Moreover, special attention should be given to reciprocal experimental designs so that “home” versus “away” effects can be explicitly evaluated. Of course, choices about the scale of infections experiments should be carefully weighed against the specific questions of interest to minimize unnecessary animal sacrifices.

## Conclusions

Our study highlights the importance of publishing results from atypical experimental outcomes. Investigating anomalies that arise during infection experiments can lead to important and unexpected insights into host-pathogen dynamics. While the unexpected Bd-positivity of the *R*. *catesbeiana* control group in our experiment lead to our results being relegated to the file drawer for many years, applying a new Bd genotyping provided insight on dynamics of infection, and co-infection, in this system. Applying new methods to old samples or datasets can help resolve ambiguities and answer previously intractable questions. We argue that more experimental anomalies should be published to serve as a resource for meta-analyses, methodological improvements, and novel contributions to wildlife disease research.

## Supporting information

S1 File Methods and results for ELISA. Description of methods used for ELISA. Table A shows results from ELISA. Samples were collected at three time points during the experiment. None of the treatment groups were significantly different than the negative control (one-tailed t-test α = .01) and all were significantly different from the positive control (one-tailed t-test p-value < .01).
(DOCX)
