# Supplementary material for: Opening the file drawer: Unexpected insights from a chytrid infection experiment
Source: PLoS One. 2018 May 9;13(5):e0196851. doi: 10.1371/journal.pone.0196851 (PMC5942794; doi:10.1371/journal.pone.0196851)
Supplement: S1 File — Description of methods used for ELISA. Table A shows results from ELISA. Samples were collected at three time points during the experiment. None of the treatment groups were significantly different than the negative control (one-tailed t-test α = .01) and all were significantly different from the positive control (one-tailed t-test p-value < .01). (DOCX) [file pone.0196851.s001.docx]

Supplementary Materials

**ELISA METHODS**

For the ELISA, we prepared the primary antibody (rabbit anti-*R. muscosa* antibody) by dilution to 1:500 in blocking buffer (APBS with 0.5% bovine serum albumin (BSA) and 0.1% Tween 20 (ABT)), and the secondary antibody (goat anti-rabbit antibody) by dilution to 1:5000 in blocking buffer. We coated the wells of a 96-well plate with pelleted, actively growing Bd cells and fixed the cells with 0.25% glutaraldehyde in APBS. We washed the plates five times with ABT before adding 50µl of 1:100 diluted frog serum. During this step positive control wells received polyclonal rabbit anti-Bd serum diluted to 1:100 in blocking buffer and the negative control wells received only blocking buffer. We incubated the plates for 90 minutes, washed the contents with ABT, and then added the 50 µl of the diluted rabbit anti-*R. muscosa* antibody. Positive control wells did not receive antibodies, but were kept wet with 50 µl ABT. Again, we incubated the plates for 90 minutes then washed the contents with ABT. We then added 50 µl goat anti-rabbit antibody (conjugated to horseradish peroxidase (HRP) and diluted in ABT) to each well. After a final incubation at room temperature for 90 minutes, we washed the plate five times and allowed it to dry. We then added 200µl of ABTS substrate (Southern Biotech, Birmingham, Alabama, USA) to each well and incubated for 60 minutes. We stopped the reaction by adding 50 µl 2M H_2_SO_4_ to all wells containing substrate and measured the optical density (OD) at 450 nm using a Bio-Rad 680 Microplate Reader (Bio-Rad, Hercules, California, USA). We used Student’s t-tests to compare mean antibody concentrations (OD) at different sampling points.

**ELISA RESULTS**

Table A: Results from ELISA. Samples were collected at three time points during the experiment. None of the treatment groups were significantly different than the negative control (one-tailed t-test α = .01) and all were significantly different from the positive control (one-tailed t-test p-value < .01).

|  |  | Optical Density [*standard deviation*] | | |
| --- | --- | --- | --- | --- |
| **Treatment Group** | **N** | **Pre** | **Day 30** | **Day 65** |
| *R. muscosa* control | 4 | 0.170 [*.028*] | 0.163 [*.024*] | 0.159 [*.028*] |
| *R. muscosa* Bd-exposed | 4 | 0.157 [*.032*] | 0.161 [*.024*] | 0.169 [*.041*] |
| *R. catesbeiana* control | 6 | 0.161 [*.017*] | 0.147 [*.017*] | 0.168 [*.023*] |
| *R. catesbeiana* Bd-exposed | 2 | 0.177 [*.019*] | 0.176 [*.007*] | 0.175 [*.019*] |
| Negative control | 2 | 0.223 [*.020*] | | |
| Positive control | 2 | 1.379 [*.094*] | | |
